# Microfluidic System to Analyze the Effects of Interleukin 6 on Lymphatic Breast Cancer Metastasis

**DOI:** 10.3389/fbioe.2020.611802

**Published:** 2021-02-15

**Authors:** Hyeon-Yeol Cho, Jin-Ha Choi, Kyeong-Jun Kim, Minkyu Shin, Jeong-Woo Choi

**Affiliations:** ^1^Department of Bio and Fermentation Convergence Technology, Kookmin University, Seoul, South Korea; ^2^Interdisciplinary Program for Bio-Health Convergence, Kookmin University, Seoul, South Korea; ^3^Department of Chemical and Biomolecular Engineering, Sogang University, Seoul, South Korea

**Keywords:** lymph vessel, cancer metastasis, epithelial-mesenchymal transition, angiogenesis, circulating tumor cells, microfluidics

## Abstract

Metastasis is the primary cause of a large number of cancer-associated deaths. By portraying the precise environment of the metastasis process *in vitro*, the microfluidic system provides useful insights on the mechanisms underlying cancer cell migration, invasion, colonization, and the procurement of supplemental nutrients. However, current *in vitro* metastasis models are biased in studying blood vessel-based metastasis pathways and thus the understanding of lymphatic metastasis is limited which is also closely related to the inflammatory system. To understand the effects of inflammatory cytokines in lymphatic metastasis, we developed a three-channel microfluidic system by mimicking the lymph vessel-tissue-blood vessel (LTB) structure. Based on the LTB chip, we successfully confirmed the inflammatory cytokine, interleukin 6 (IL-6), -mediated intercellular communication in the tumor microenvironment during lymphatic metastasis. The IL-6 exposure to different subtypes of breast cancer cells was induced epithelial-mesenchymal transition (EMT) and improved tissue invasion property (8-fold). And the growth of human vein endothelial cells toward the lymph vessel channel was observed by VEGF secretion from human lymphatic endothelial cells with IL-6 treatment. The proposed LTB chip can be applied to analyze the intercellular communication during the lymphatic metastasis process and be a unique tool to understand the intercellular communication in the cancer microenvironment under various extracellular stimuli such as inflammatory cytokines, stromal reactions, hypoxia, and nutrient deficiency.

## Introduction

Cancer metastasis is a phenomenon involving the spread and proliferation of the primary cancer cells into secondary sites through the circulatory system, including lymph and blood vessels (Gupta and Massague, 2006). Cancer cells are heterogeneous and have different migration properties based on their phenotypes, which are strongly associated with malignancy (Brabletz et al., 2005). However, for the progress of metastasis from a benign cancer cell, epithelial-mesenchymal transition (EMT) and angiogenesis are necessary for the early stages (Kang and Massague, 2004). In the primary cancer microenvironment, EMT can be induced by various extracellular stimuli such as stromal reactions, hypoxia (Matsuoka et al., 2013), nutrient deficiency (Recouvreux et al., 2020), and inflammatory cytokines (Muller et al., 2001). Epithelial cells undergo a change in their cell polarities, cytoskeletal systems, and cell-cell adhesion properties, and transition to mesenchymal cells with migratory and invasive properties (Yilmaz and Christofori, 2009). Subsequently, these cells present as circulating tumor cells (CTC), circulate the blood vessels and lymph nodes, and metastasize to other organs by the mesenchymal-epithelial transition (MET), which is the opposite of the EMT process (Banyard and Bielenberg, 2015). Subsequently, new blood vessels are generated in the anchored cancer cells by a process called angiogenesis, and the nutrients and oxygen necessary for their maintenance and proliferation are supplied to these cells (Folkman, 2002). This is one of the primary reasons why cancer is a very serious disease and difficult to treat. Therefore, this study is necessary to identify effective cancer therapies involving the prevention and elimination of metastasis.

EMT and angiogenesis are very important steps in the development and progress of cancer metastasis (Panchy et al., 2020). To study these phenomena, several researchers have used diverse model systems, including *in vivo* animals (Pereira et al., 2018) and *in vitro* cell culture models (Webb et al., 2017; Meng et al., 2019). Among these, animal surrogate models have been widely used (Pulaski and Ostrand-Rosenberg, 2000). However, they are extremely complex, and it is difficult to independently and selectively control each contributing factor and analyze the cause and effect. Further, the inherent differences between animal and human species result in inevitable failures in the prediction of pathophysiological progress (Martine et al., 2017). Conversely, *in vitro* models have the advantage of presenting simple and similar analyses owing to the use of human cells (Cho et al., 2018; Bu et al., 2019; Kim et al., 2020). In particular, the microfluidic model can present a system more similar to the human body compared to an animal model, owing to properties like the fluidic flow and shear stress (Sontheimer-Phelps et al., 2019; Mondadori et al., 2020). Moreover, since it is possible to cultivate two or more human cells simultaneously, it can be used to demonstrate intercellular communication, which is a critical point of research in cancer metastasis, including EMT and angiogenesis (Li et al., 2018; Lee et al., 2020). For these reasons, the microfluidic culture system has been used to study EMT and observe metastasis. Moreover, it is relatively easy to develop a microfluidic system for monitoring EMT and mesenchymal-epithelial transition (MET) during metastasis by replicating the capillary blood vessels and EMT-induced circulating tumor cells (CTC).

**Figure 1:**
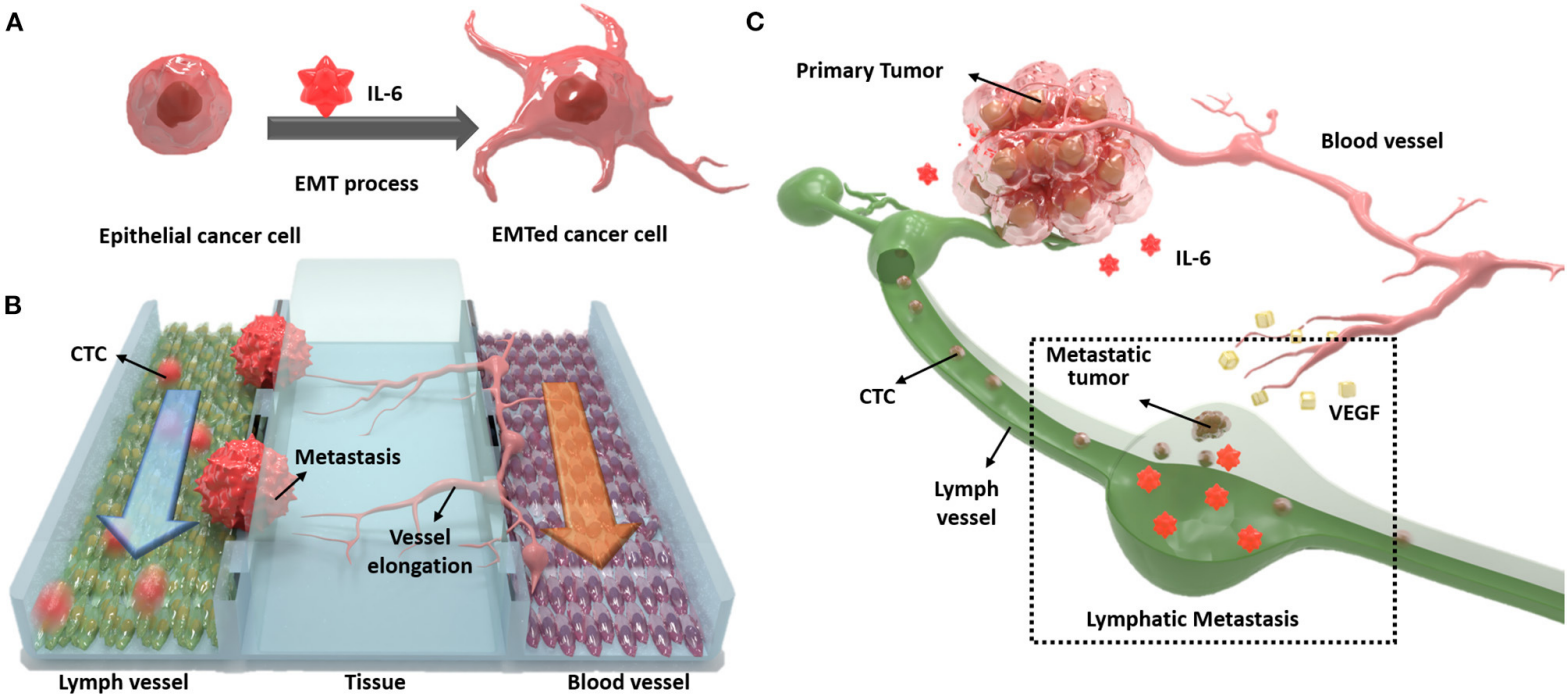
Schematic diagram of the lymph vessel-tissue-blood vessel (LTB) chip. **(A)** Interleukin 6 (IL-6) induces epithelial-mesenchymal transition (EMT) of cancer cells and enhances metastatic properties. **(B)** LTB chip is designed to mimic the metastasis process of circulating tumor cells (CTCs) in the lymph vessel channel including colonization and recruitment of blood vessel cells. **(C)** Illustration of *in vivo* lymphatic metastasis process. The dash-line box indicates where the LTB chip mimics.

In this study, we designed a novel three-channel microfluidic chip for lymphatic metastasis by mimicking the lymph vessel-tissue-blood vessel (LTB) structure (Figure 1). The LTB chip consisted of a microfluidic chip that illustrated inflammatory cytokine-mediated EMT and angiogenesis in breast cancer, which involves the metastasis of tumor cells through the lymph vessel (Figure 1A). The lymphatic endothelial cells, which were critically affected by the CTCs, secreted vascular endothelial growth factor (VEGF), which induced angiogenesis and promoted breast cancer metastasis by promoting tumor invasiveness (Gong et al., 2019). The current *in vitro* microfluidic model of metastasis has focused on blood vessel-mediated metastasis. Recently, several microfluidic chips were developed to monitor the communication between cancer and lymph node, but this system was not showed the interaction with blood vessels (Shim et al., 2019). Also, a microfluidic model for analyzing cooperative effects of vascular angiogenesis and lymphangiogenesis was recently reported but this system was focused on direct interaction between blood and lymph vessel channels, not the effects of inflammatory cytokines (Osaki et al., 2018). Our newly designed LTB chip contained blood and lymph vessel channels to induce angiogenesis as a result of the EMT process of cancer cells (Figure 1B). Using this microfluidic chip platform, we reproduced and analyzed metastasis through the interaction between IL-6-treated cells and lymph and blood vessel channels (Figure 1C). This is the first study to replicate the metastasis process from EMT to angiogenesis in the lymph and blood vessel channels.

## Materials and Methods

### Materials

The silicone elastomer (polydimethylsiloxane, PDMS) was purchased from Dow Corning (Midland, MI), and the Matrigel matrix was purchased from Corning (Bedford, MA). IL-6 and other chemicals were purchased from Sigma-Aldrich (St. Louis, MO).

### Fabrication of the LTB Chip

The microchannel structure of the LTB chip was fabricated using the replica molding technique. First, we designed the device *in silico* using a computer-aided design (CAD) program (Mun et al., 2020). We created a positive relief 100 μm pattern on a silicon wafer by cleaning and dehydrating a 6-inch wafer and coating it uniformly with SU-8, the photoresist, using a spin coater. Subsequently, the coated wafer was pre-baked at 65°C on a level hot plate until the solvent evaporated, and the density of the SU-8 film increased. The coated wafer was then exposed to UV light using a transparent photomask to obtain a positive relief microfluidic channel. The wafer was baked at 95°C on a hot plate for selective cross-linking, and the microfluidic channel was developed using a photoresist developer. Finally, the wafer was briefly rinsed with isopropyl alcohol and dried using nitrogen gas.

A negative replica of the mold was made with PDMS by attaching the disposable chamber to the mold. The PDMS solution was cured in a convection oven at 70°C for 1 h, and the replica was peeled from the mold. Subsequently, the basement (cover glass) was coated with 20 μm thick PDMS and pre-cured for 10 min to establish a stable and strong connection between the replica and the basement. The replica was attached to a PDMS-coated basement and cured for 2 h. To establish a stronger connection between the replica and the basement, air oxygen plasma was treated (5 min for replica and basement) in the attachment step. After, a hole was introduced from the top to the basement, to create an inlet and outlet. The fabricated LTB chip was then autoclaved and sterilized at 120°C for 30 min, washed with deionized water (DW), and stored under dry conditions.

The matrigel was injected into the middle channel of the autoclaved LTB chip to make a hydrogel block in the chip, and incubated at 37°C for 1 h, under humid conditions, to facilitate gelation.

### Cell Culture

Three breast cancer cell lines (MCF-7, MDA-MB-231, and SK-BR-3) were obtained from ATCC (Manassas, VA). All cell culture media and supplements were obtained from Invitrogen (Carlsbad, CA) unless otherwise noted. MDA-MB-231 cancer cell lines were grown in RPMI 1640 media, while MCF-7 and SK-BR-3 cells were grown in Dulbecco's Modified Eagle Medium (DMEM). All media were supplemented with 10% fetal bovine serum (FBS) and 1% Penicillin-Streptomycin. Cells were grown at 37°C and 5% CO_2_ in a TC-grade Petri dish (BD Biosciences, San Jose, CA). The stem-like human breast cancer cell (S-HBCC) line and culture media were purchased from Celprogen (36102-29, Torrance, CA). These cells were grown at 37°C and 5% CO_2_ in a TC-grade Petri dish (BD Biosciences, San Jose, CA). The media was renewed every 3 days for all three breast cancer cell lines and every 2 days for the S-HBCC. The human umbilical vein endothelial cells (HUVECs, ATCC) and human lymphatic endothelial cells (HLECs) were purchased from ScienCell Research Laboratories and maintained in endothelial cell medium (ECM, Sciencell, San Diego, CA) at 37°C and 5% CO_2_. A matrigel coated culture plate was used to maintain HUVECs and HLECs coated with matrigel (1:20) in serum-free media for 1 h, to create a high-affinity surface with endothelial cells.

### Cell Culture in the LTB Chip

Prior to the injection of endothelial cells, the inside of the LTB chip was filled with a fresh endothelial cell medium. To form a HUVECs and HLECs layer on the side of the hydrogel block in the LTB chip, each cell solution (300 μL, 1 × 10^7^ cells/ml) was slowly added to the 90° rotated LTB chip and incubated for 12 h to let them attached on the surface of hydrogel block. Following the immobilization of the endothelial cells, fresh endothelial cell medium was added at a constant flow rate of 10 μL/min for 48 h, to form an endothelial cell layer on the side surface of the hydrogel block. Breast cancer cells were injected into the endothelial layer of the LTB chip with a flow rate of 5 μL/min for 6 h, and fresh cancer cell media was added continually. The entire process of cell culture and cell injection was performed in an incubator at 37°C and 5% CO_2_. For the tracking purpose, breast cancer cells were labeled with membrane staining dyes (Blue: BioTracker 400 Cytoplasmic Membrane Dye, SCT109, Sigma-Aldrich, Red: DiI, D282, ThremoFisher Scientific).

### Image Acquisition

All images were acquired using a confocal laser scanning microscope (Zeiss LSM 710; Carl Zeiss, Oberko, Germany) with a 16-bit monochromatic CCD (Orca R2; Hamamatsu Photonics, Shizuoka, Japan), under the 10x objective lens (LUCPlan FLN, NA: 0.45; Olympus, Tokyo, Japan). Fluorescent images were acquired in three separate channels, sequentially, to minimize potential cross-talk effects. The entire surface of the chip was scanned using a motorized stage to identify CTCs, based on cell morphology and immunofluorescent staining.

### Quantification of VEGF Protein Secreted From HLEC

Cellular supernatants were harvested from HLEC and assayed for the amount of vascular endothelial growth factor (VEGF) secreted using the VEGF Human ELISA Kit (Abcam Cambridge, MA). A total number of 20,000 cells were seeded in 96-well plates and allowed to adhere overnight. Subsequently, the cells were thoroughly rinsed with 1X PBS, and 200 μL of fresh RPMI complete media with IL-6 protein (100 ng/mL) was added to the cells. Cell cultures were incubated for 48 h, and the soluble supernatants were collected and assayed for VEGF protein, by ELISA, following the manufacturer's instructions.

### Invasion Assay

The cell invasion ability was determined using a BioCoat Matrigel invasion chamber (BD Biosciences, Bedford, MA). The chamber membrane filter (pore size of 8 μm) was coated with a BD Matrigel Basement Membrane Matrix (BD Biosciences). The upper chamber was loaded with 25,000 cells in 0.5 mL of serum-free medium, and the lower chamber was filled with 0.75 mL of serum-containing medium. Following 22 h of incubation at 37°C and 5% CO_2_, the non-invading cells on the upper surface of the membrane were removed using cotton swabs. The invading cells on the lower surface of the membrane were washed in PBS, fixed in paraformaldehyde, and stained with Hoechst 33342. For each membrane filter, the number of invading cells in 10 randomly selected fields were counted under a confocal laser scanning microscope (Zeiss LSM 710; Carl Zeiss, Oberko, Germany).

### RNA Extraction and RT-PCR Analysis

Total RNA was isolated using TRIzol (Invitrogen), and cDNA was synthesized from 2 μg of total RNA using the SuperScript III First-Strand Synthesis Kit (Invitrogen). Subsequently, PCR was carried out using the AccuPower PCR-Premix (Bioneer, Daejeon, Korea). The primer sequences and reaction conditions are listed in Supplementary Table 1. The relative band intensities were determined using a spectrum imaging system (UVP, Upland, CA, USA). The target mRNA levels were normalized to the control condition after the normalization with a signal obtained for glyceraldehyde-3-phosphate dehydrogenase (GAPDH) mRNA expression.

### Statistical Analysis

All experiments were repeated at least five times. Data are shown as means ± standard deviations. Statistical significance was determined by Welch's *t*-test with differences considered statistically significant at a value of *P* < 0.05.

## Results

### Characterization of IL-6-Treated Breast Cancer Cells

To mimic the cancer metastasis environment, a microfluidic chip was designed *in situ*. The IL-6-treated cancer cells (considered as CTC), colonization, and invasion were monitored using three parallel channels, lymphatic vessel channel, blood vessel channel, and the extracellular matrix between the channels. The middle channel was filled with hydrogel to replicate the three-dimensional extracellular matrix with a tissue. Lymphatic and vascular endothelial cells were immobilized to form a layer on the vertical side of the hydrogel channel in the LTB chip. Prior to the fabrication of the microfluidic chip, EMT was induced to the breast cancer cells by treating various kinds of cytokines and microRNAs that generally exist in the cancer environment and induce the EMT process. These include the transforming growth factor-β1 (TGF-β1) for triggering EMT-associated pathways (Pang et al., 2016), microRNA to regulate pro-metastatic genes (Bullock et al., 2012), and interleukin-6 (IL-6) to downregulate the expression of epithelial markers (Sullivan et al., 2009). After the generation of two endothelial cell layers with the hydrogel, the IL-6-treated breast cancer cells were injected into the lymphatic channel to emulate the cancer metastasis process in the microfluidic system. In this study, we used IL-6 to change the properties of various breast cancer cells from those of epithelial cells to mesenchymal cells.

**Figure 2:**
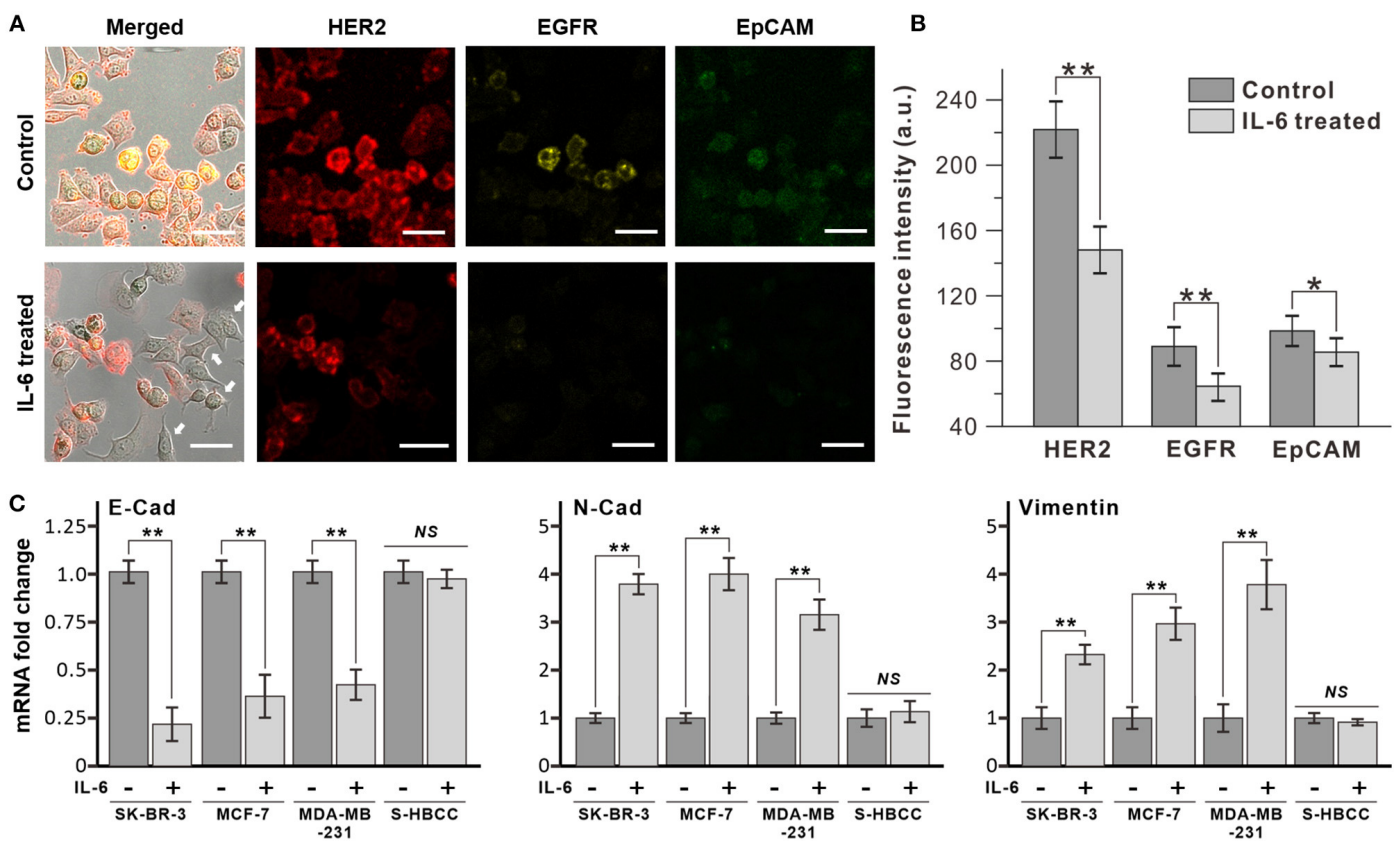
Decrease of epithelial marker expression by interleukin 6 (IL-6). **(A)** Immunofluorescence analysis of breast cancer cell (SK-BR-3) with IL-6 treatment. After 2 days, dramatic morphological changes were shown with decrease surface marker expression (bottom row, IL-6 treated) compared to untreated condition (upper row, control). White arrows indicate cells that have morphology change and lower surface marker expression. **(B)** Comparison of surface marker signal intensity with IL-6 treatment. **(C)** RT-PCR results of EMT-related gene expression on different subtypes of breast cancer cells. The data represent means ± standard deviations (SD) of five different experiments (*p < 0.05, **p < 0.01). RT-PCR results are normalized with control after the normalization with GAPDH.

The exposure of breast cancer cells to IL-6 induced dramatic morphological changes in the cells, from the epithelial to the mesenchymal structure. In Figure 2A, HER-2 positive human breast cancer cells (SK-BR-3) were ramblingly stretched and spread out, while the untreated cells presented a round shape. Apart from the morphological changes, the expression of surface markers on the IL-6-treated cancer cells almost reduced to half (Figure 2B). This alteration in the expression of surface markers indicates an inherent change in its cellular properties. This was confirmed by measuring the expression levels of three different surface markers, HER2, EGFR, and EpCAM, expressed on breast cancer cells, by immunofluorescence analysis (Figure 2A). Interestingly, following IL-6 treatment, only the non-stretched cells showed similar intensity in the expression of specific surface markers.

The morphological changes in SK-BR-3 cells were analyzed by reverse transcription-PCR (RT-PCR) through the measurement of representative epithelial and mesenchymal marker expressions (Figure 2C). After 48 h IL-6 exposure, the SK-BR-3 cells showed dramatic downregulation of E-cadherin and upregulation of N-cadherin which is a representative pattern of the EMT process. Moreover, these two proteins are closely related to cell adhesion, motility as well as morphology. Similarly, other subtypes of breast cancer cell lines including MCF-7 (luminal type), and MDA-MB-231 (basal type) also showed a decrease of E-cadherin and an increase of N-cadherin with Vimentin with IL-6 exposure while S-HBCC (cancer stem cell type) was not showed significant change. Consequently, IL-6 induced the EMT process to various subtypes of breast cancer cells except for the stem cell populations.

**Figure 3:**
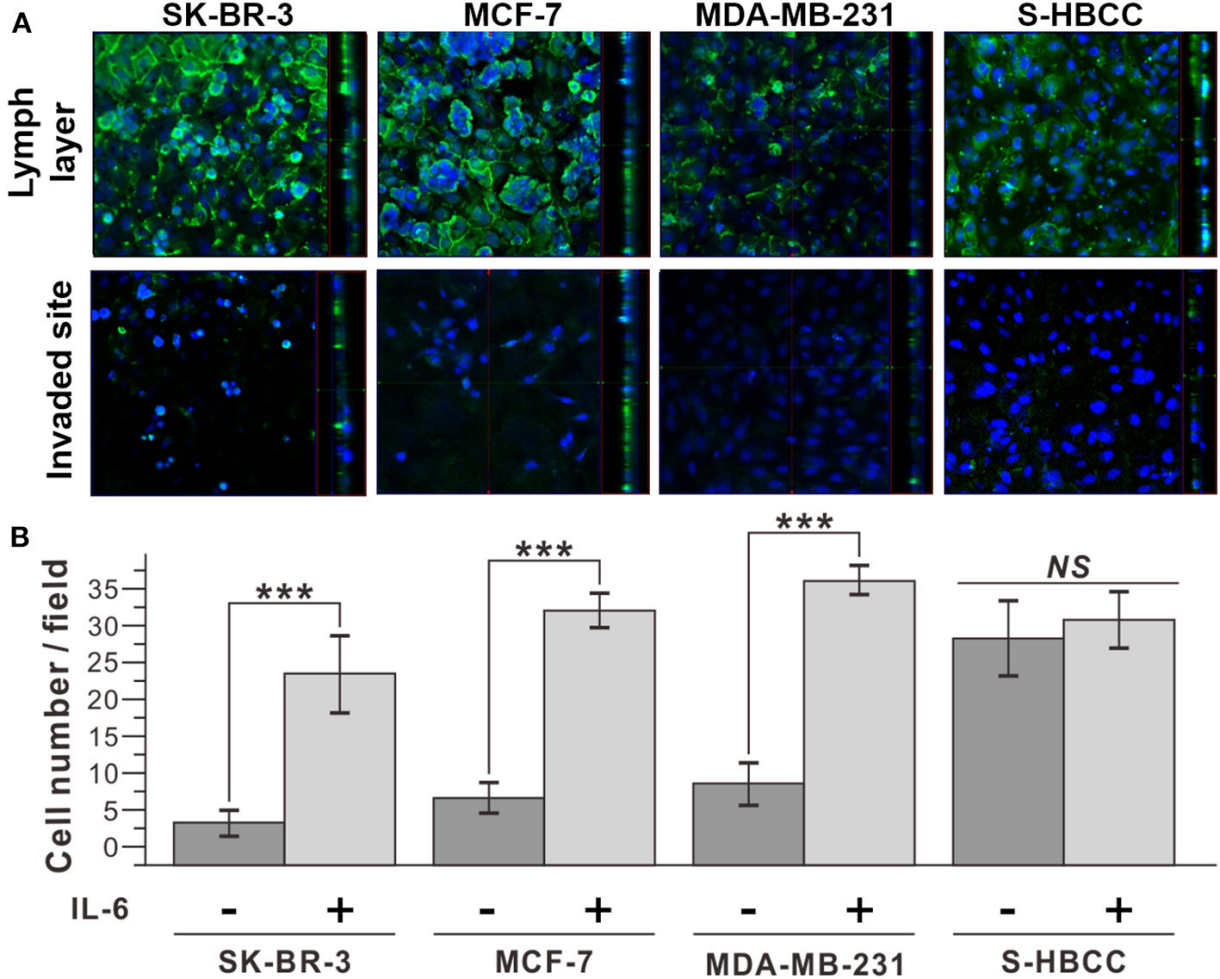
Improved invasiveness of IL-6-treated breast cancer cells with IL-6. **(A)** Confocal microscope images of IL-6 treated breast cancer cells. The upper row shows the top side of the hydrogel block with the HLEC layer and the lower row shows the bottom side of the hydrogel block. (Green: CD31, Blue: Nucleus) **(B)** Cell counting analysis after cell penetration through the hydrogel. IL-6-treated cells were obtained by IL-6 treatment for 2 days. The data represent means ± standard deviations (SD) of five different experiments (***p < 0.001).

Additionally, we conducted an invasion assay to confirm the metastatic property of IL-6-treated cells following treatment with IL-6. To determine whether IL-6 exposure functionally enhanced the invasive capacity of breast cancer cells, we used a cell invasion chamber composed of Transwells and embedded with hydrogel. Prior to the seeding of breast cancer cells, HLECs were immobilized on the top side of the hydrogel in the invasion chamber to mimic the actual human lymph vessel. We found that the control and IL-6-treated breast cancer cells exhibited different degrees of invasiveness through the lymphatic, layered-hydrogel in the presence of FBS as a chemoattractant (Figure 3A). Every subtype of the IL-6-treated breast cancer cell except the S-HBCC was significantly (*P*-value < 0.01) more invasive and attained over a 5-fold increase compared to the control cells (SK-BR-3: 8-fold; MCF 7: 4.97-fold; and MDA-MB-231: 4.35-fold) (Figure 3B). The S-HBCCs showed high invasiveness from both control and IL-6 treated conditions while there were no significant changes, which is concordant with the lack of change in E-cadherin expression at a low level (Supplementary Figure 1). Therefore, IL-6 can induce the EMT process on various subtypes of breast cancer cells, except the stem-like subtype, and also enhances the invasiveness of tumor cells.

### Inducing VEGF Secretion From HLECs for Cancer Metastasis

**Figure 4:**
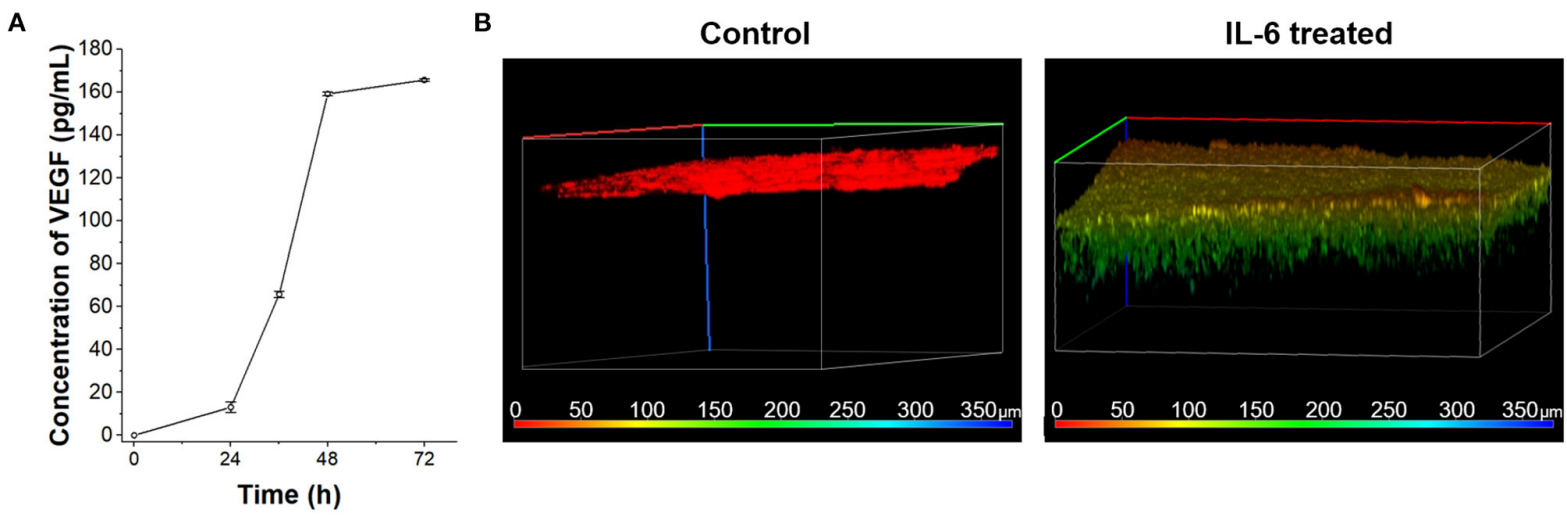
VEGF secretion of HLECs by IL-6 treatment. **(A)** Time-dependent VEGF secretion from HLECs under the IL-6 (100 ng/mL) contained HLEC maintain media. **(B)** Recruitment of blood vessel cells (HUVECs) growth test toward control (HLEC cultured media without IL-6 treatment) and IL-6 treated (HLEC cultured media with IL-6 treatment) conditions. The pseudo color was applied to show the depth profile.

According to the *in vivo* research, the interaction between IL-6 and lymphatic endothelial cells in lymphatic metastasis of breast cancer were induced angiogenesis (Lee et al., 2014a,b). To confirm the IL-6-mediated angiogenesis process in a human cell-based LTB chip can mimic a lymph system in the mouse model, 100 ng/mL IL-6 was treated with HLECs in culture media (Figure 4A). Among various angiogenic factors, VEGF was selected as a representative marker to follow *in vivo* research (Lee et al., 2014a). The concentration of secreted VEGF was analyzed with the ELISA assay. As expected, secretion of VEGF was stimulated with IL-6 treatment and linearly increased in a time-dependent manner while control condition didn't show VEGF secretion (data not shown). However, the amount of VEGF in media was saturated after 48 h of IL-6 exposure because of the consumption (Gao et al., 2017) or fast degradation (half-life 15.5 h, Kuribayashi, 2018) of IL-6 in batch culture condition.

The VEGF secreted from the lymphatic endothelial cell recruited the blood vessels from pre-existing vessels. To verify the angiogenic effect of cytokines secreted from HLEC with IL-6 treatment on HUVECs, a vascular endothelial cell, the Transwell platform-based density gradient system was utilized (Figure 4B). HUVECs were seed on the top side of a hydrogel block in Transwell. The area outside the Transwell was filled with HLEC cultured media with IL-6 for 2 days and HUVEC seeded side was filled with HUVEC culture media to eliminate the nutrient-taxis effects. After 5 days, HUVECs were vertically grown more than 100 μm toward the side containing the HLEC culture media while the control (HLEC cultured media for 2 days without IL-6) didn't show any distinctive change. It indicates that the level of VEGF secreted from HLECs was sufficient to communicate with HUVECs. As similar to the saturation of VEGF secretion in a batch culture condition, the vertical growth speed of HUVECs was also decreased with the consumption of VEGF (Jeong et al., 2011). Both issues with batch culture conditions can be overcome with the continuous supply of substrates (IL-6) by using the microfluidic chip system.

### Monitoring of IL-6-Mediated Cancer Metastasis in the Microfluidic Chip

**Figure 5:**
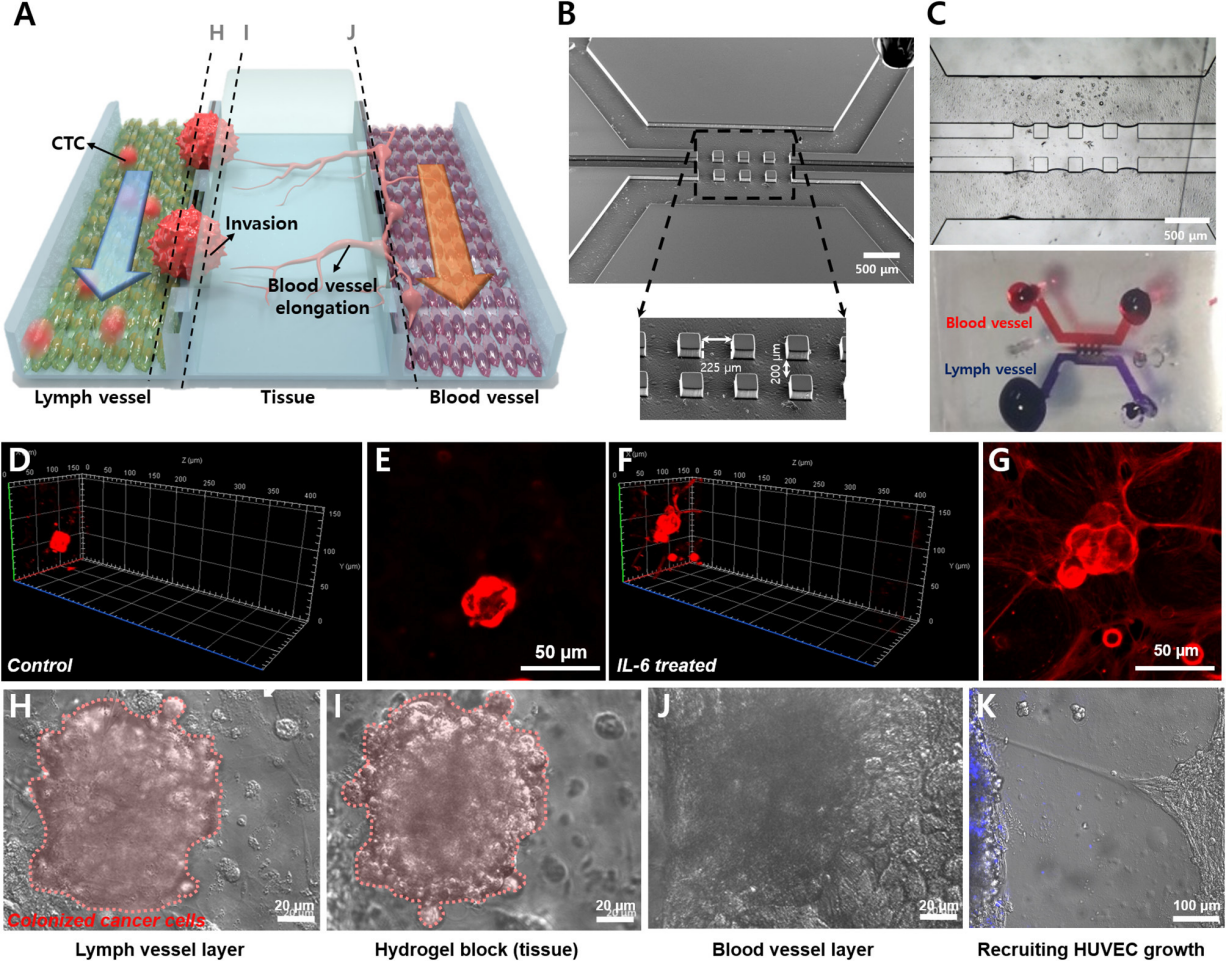
Demonstration of lymphatic metastasis process in LTB chip. **(A)** Schematic diagram of the lymphatic metastasis process in LTB chip. **(B)** SEM image of the three-channel structure of LTB chip. Scale bar: 500 μm. **(C)** Hydrogel block formation in the middle channel. Hydrogel block showed prevention of direct connection between lymph channel and blood channel while small molecules can slowly diffuse to each side. **(D–G)** Colonization of control **(D,E)** and IL-6 treated SK-BR-3 cells **(F,G)** on the endothelial cell seeded hydrogel block. For **(D)** and **(F)**, left side is lymph vessel channel and right side is blood vessel channel. Red: membrane dye (DiI) labeled cancer cells. **(D)** 3D confocal image of IL-6 non-treated SK-BR-3 and **(E)** 2D image on the HLEC layer. **(F)** 3D confocal image of IL-6 treated SK-BR-3 and **(G)** 2D image on the HLEC layer. **(H–J)** Microscopic image of colonized breast cancer cell in LTB chip (vertical section images). **(H)** Colonized breast cancer cell cluster on the surface of HLEC layer (pink color labeled (pseudo color), left side of hydrogel block). **(I)** Tissue penetration of cancer cells. **(J)** The surface of HUVEC layer (right side of the hydrogel block). **(K)** Growth of HUVECs toward lymph channel (left). Blue: membrane dye (BioTracker 400) labeled cancer cells.

The LTB chip was fabricated with three-channel structures consisting of a lymph vessel channel, extracellular matrix, and blood vessel channel (Figure 5A). Three squared pillars separated the three channels and the middle channel was loaded with hydrogel (Figure 5B). Figure 5C demonstrates the three-dimensional matrix in the middle layer and the flow of fluid in the blood and lymph vessel channels independently, with permeation through the hydrogel. Two different endothelial cells were immobilized separately, HLECs and HUVECs. After the formation of an endothelial layer on the side of the hydrogel block, the breast cancer cells were injected for 6 h with IL-6 containing fresh media, to induce the EMT process on the cancer cells and facilitate VEGF secretion from HLECs. The flow rates of the fluid in the blood and lymph vessel channels were set differently to match *in vivo* conditions (lymph vessel channel: 5 μL/min, blood vessel channel: 15 μL/min). To validate the effects of IL-6 on HLECs and cancer cells in the microfluidic system, we obtained optical images of the cells in each fluidic channel. As expected, the injected breast cancer cells were attached to the sidewall of the lymph channel and colonized as a solid tumor (Figures 5D–G). Even though cancer cells were formed clusters in both control and IL-6 treated conditions, but invasion behavior was different. When breast cancer cells were colonized without IL-6, the shape of colonies was similar to spheroids with a plane surface (Figures 5D,E). Differently, IL-6 treated breast cancer cell colonies were stretched out to surrounding areas with sharp tip ends (Figures 5F,G). However, after 1 week from the colonization, the size of the colony reached around 100 μm and saturated (Figures 5H–J). Subsequently, the HUVECs were grown toward the lymphatic side, forming the shape of a tip (Figure 5K). Additionally, we found that when HUVECs were grown toward the lymph vessel channel, the tip end was headed to the cancer cell clustered site (blue labeled). It was known that breast cancer cell-HLEC co-culture induces higher secretion (up to 600%) of VEGF from HLECs with both SK-BR-3 (Tawada et al., 2014) and MDA-MB-231 cells (Lee et al., 2014b). Consequently, we successfully mimicked cancer metastasis, including the EMT process of breast cancer cells and angiogenesis of blood and lymph vessel channels in the microfluidic device.

## Discussion

In this study, we fabricated a microfluidic chip replicating the blood and lymph vessel channels in the human body and monitored the effects of inflammatory cytokine, IL-6, on breast cancer metastasis process at the *in vitro* condition. EMT was induced in the breast cancer cells by IL-6 treatment. Every subtype of breast cancer cell showed upregulation of mesenchymal-related gene profile, morphological changes as shown in Figure 2. The invasion assay revealed that the IL-6-treated breast cancer cells presented higher transvascular invasiveness compared to the untreated cells as shown in Figure 3. To fabricate the LTB chip, a three-channel microfluidic was designed for *in situ* monitoring of cancer cell colonization and invasion. The middle channel was filled with collagen-based hydrogel to replicate the tissues *in vivo*. HLECs and HUVECs were seeded to form a layer on the side of the hydrogel in the LTB chip. Subsequently, the IL-6-treated breast cancer cells were injected along with IL-6-containing media into the LTB chip, and the injected cells were monitored using a confocal microscope. The IL-6-treated breast cancer cells successfully immobilized and colonized on the HLEC layer and began invading into the inner side of the hydrogel layer. Subsequently, the HLECs were stimulated by IL-6 to secrete VEGF, which caused the HUVECs to grow toward the cancer cell clusters near the lymph channel as shown in Figures 4, 5. Even though the LTB chip showed recruitment of blood vessel cells in the chip, morphology of HUVECs in the angiogenesis process has to form the microvascular structure (Ko et al., 2019; Sewell-Loftin et al., 2020). It may happen by forming a small niche between the hydrogel block and bottom substrate during the gelation process. By addressing recent 3D printing-based *in vitro* metastatic model system, the current issue of our LTB chip can be covered and improved to closely mimicked *in vivo* system (Meng et al., 2019).

To this end, effects of IL-6 on lymphatic metastasis of breast cancer cell was mimicked and analyzed with a newly developed LTB chip, therefore, it can be utilized as an analysis platform for understanding the effects of various inflammatory cytokines on metastasis. Moreover, the LTB chip will offer a new platform to understand the interaction between the tumor microenvironment and the lymph system. The improvement of knowledge of subtype-specific tumor behavior in the lymph system will provide crucial information for early diagnosis, prognosis monitoring, and personalized therapy for breast cancer patients.

## Data Availability Statement

The original contributions presented in the study are included in the article/Supplementary Material, further inquiries can be directed to the corresponding author.

## Author Contributions

H-YC and J-WC designed the experiment. H-YC and K-JK designed and fabricated the microfluidic device. H-YC, J-HC, MS, and J-WC collected and analyzed the results. All authors collaboratively wrote the manuscript.

## Conflict of Interest

The authors declare that the research was conducted in the absence of any commercial or financial relationships that could be construed as a potential conflict of interest.
