# Supplementary material for: Microfluidic System to Analyze the Effects of Interleukin 6 on Lymphatic Breast Cancer Metastasis
Source: Front Bioeng Biotechnol. 2021 Feb 15;8:611802. doi: 10.3389/fbioe.2020.611802 (PMC7917128; doi:10.3389/fbioe.2020.611802)
Supplement: Supplementary file 1 [file Data_Sheet_1.docx]

Microfluidic system to analyze the effects of Interleukin 6 on lymphatic breast cancer metastasis

Hyeon-Yeol Cho^1,2,†^, Jin-Ha Choi^3,†^, Kyeong-Jun Kim^3^, Minkyu Shin^3^, Jeong-Woo Choi^3,*^

^1^Department of Bio & Fermentation Convergence Technology, Kookmin University, Seoul, South Korea

^2^Interdisciplinary Program for Bio-health Convergence, Kookmin University, Seoul, South Korea

^3^Department of Chemical & Biomolecular Engineering, Sogang University, Seoul, South Korea

^†^Equal contribution

*** Correspondence:**Jeong-Woo Choi
jwchoi@sogang.ac.kr

Supplementary Material

**
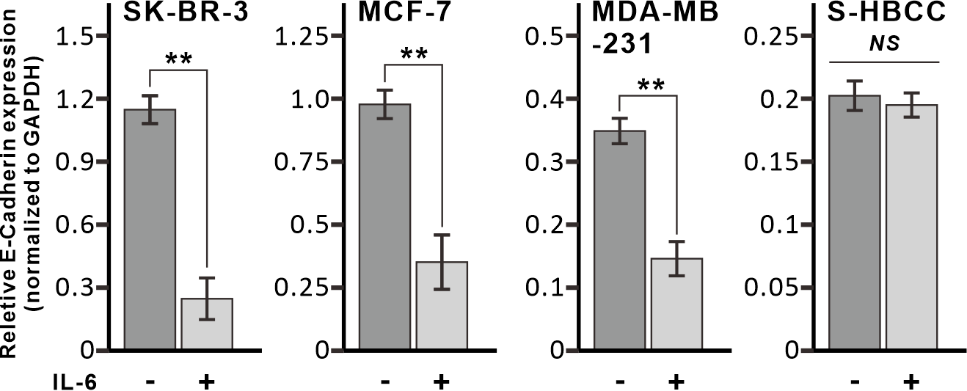
**

**Supplementary Figure 1.** RT-PCR analysis of IL-6-mediated E-cadherin expression changes on breast cancer cells without normalization to the control (-, IL-6 not treated) condition.

**Supplementary Table 1. Primers and reaction conditions for RT-PCR**

| **Gene** | **Forward (5’→3’)** | **Reverse (5’→3’)** |
| --- | --- | --- |
| GAPDH | AGCCACATCGCTCAGACACC | GTACTCAGCGCCAGCATCG |
| E-Cadherin | AGGAATTCTTGCTTTGCTAATTCTG | CGAAGAAACAGCAAGAGCAGC |
| Vimentin | AGGCAAAGCAGGAGTCCACTGA | ATCTGGCGTTCCAGGGACTCAT |
| N-Cadherin | CCTCCAGAGTTTACTGCCATGAC | GTAGGATCTCCGCCACTGATTC |
